# Changes in the molecular profiles of large-vessel vasculitis treated with biological disease-modifying anti-rheumatic drugs and Janus kinase inhibitors

**DOI:** 10.3389/fimmu.2023.1197342

**Published:** 2023-05-01

**Authors:** Kotaro Matsumoto, Katsuya Suzuki, Masaru Takeshita, Tsutomu Takeuchi, Yuko Kaneko

**Affiliations:** Division of Rheumatology, Department of Internal Medicine, Keio University School of Medicine, Shinjuku-ku, Tokyo, Japan

**Keywords:** giant cell arteritis, takayasu arteritis, biological disease-modifying anti-rheumatic drugs, janus kinase inhibitors, molecular remission

## Abstract

Giant cell arteritis and Takayasu arteritis are two types of primary large-vessel vasculitis (LVV). Although glucocorticoids (GC) are the standard treatment for LVV, the disease relapse rates are high. Recent clinical trials on biological disease-modifying anti-rheumatic drugs (bDMARDs) and Janus kinase (JAK) inhibitors have demonstrated their efficacy in reducing LVV relapse rates and GC dosages. However, the control of residual inflammation and degenerative alterations in the vessel wall remains an outstanding requirement in the clinical management of LVV. The analysis of immune cell phenotypes in patients with LVV may predict their response to treatment with bDMARDs and JAK inhibitors and guide their optimal use. In this mini-review, we focused on molecular markers, including the immune cell proportions and gene expression, in patients with LVV and in mouse models of LVV treated with bDMARDs and JAK inhibitors.

## 1 Introduction

Giant cell arteritis (GCA) and Takayasu arteritis (TAK) are two types of primary large-vessel vasculitis (LVV) characterized by a predominantly granulomatous infiltration of T cells, macrophages, and multinucleated giant cells (1). The pathophysiology of LVV is not sufficiently elucidated; however, the involvement of Th1 and Th17 immune-mediated responses and an imbalance between Th17 and regulatory T (Treg) cells have been demonstrated in LVV (2–12). Glucocorticoids (GC) are used as a standard treatment for LVV and are effective in inducing remission. However, relapse during the maintenance phase is common, and long-term GC use may be associated with substantial negative effects (13–15). GC therapy ameliorates vasculitis symptoms, and the persistence of Th1, Th17, and myeloid cells during treatment can lead to disease relapse (2–4, 16–20).

Recent clinical trials have demonstrated promising results for biological disease-modifying anti-rheumatic drugs (bDMARDs) and Janus kinase (JAK) inhibitors in the reduction of LVV relapse and tapering of GC dose. However, the response to these therapies is variable. Therefore, it is essential to identify molecular signatures that can predict treatment response and guide treatment optimization. In patients with LVV and in mouse models of LVV, analysis of immune cell phenotypes can provide insight into the molecular mechanisms of bDMARDs and JAK inhibitors and predict treatment response. In this mini-review, we investigated the longitudinal changes in immune cell phenotypes in patients with LVV and in mouse models of LVV treated with bDMARDs and JAK inhibitors.

## 2 Molecular profile alterations in LVV under treatment

The molecular profiles of patients with LVV and mouse models of LVV treated with bDMARDs and JAK inhibitors are presented in **Tables 1**, **2**, respectively. We reviewed the evidence for the following treatments: interleukin (IL)-6 receptor antagonists, tumor necrosis factor (TNF)-α receptor antagonists, IL-1 receptor antagonists, granulocyte-macrophage colony-stimulating factor (GM-CSF) receptor antagonists, and JAK inhibitors. Although there are no established animal models of LVV, studies that used IL-1Ra-deficient mice and human artery engrafted mice as LVV models were reviewed. IL-1Ra-deficient mice have autoimmune diseases similar to those in humans, including aortitis, arthritis, and skin manifestations (36, 37). Aortitis involves aortic valve wall thickening and regurgitation and resembles TAK. The human artery engrafted mouse is a model of GCA in which human temporal arteries are grafted into severe combined immunodeficiency mice (38).

**Table 1:** Molecular profiles in patients with large vessel vasculitis associated with treatments.

	GCA	TAK
IL-6 receptor antagonist	• Improved the imbalanced proportions of Th17, Tfh, and Treg (7–11) • Partially reverse Treg dysfunction (9) • Normalized the gene expression associated with M0-like monocyte, NK, Tfh, and Treg (21)	• Improved the imbalanced proportions of Th17, Tfh, and Treg (10, 11) • Less effect on the proportion of Th1 and CD8 (10, 11)
TNF-α receptor antagonist	• Not effective (22–24)	• Reduced the residual gene signature under treatment with glucocorticoids (25) • Partially reduced the proportion of CCL2-producing macrophage (26, 27)
IL-1 receptor antagonist	• Elevated IL-1 signaling pathway was associated with future relapse (25)	
GM-CSF receptor antagonist	• Reduced Th1 differentiation (28)	
JAK inhibitors	• Type I IFN signature was enriched in the aorta of LV-GCA (29) • Part of the IFN gene signature was elevated in peripheral blood and associated with disease relapse (25)	• Type I IFN signature was enriched in CD4 and CD8 lymphocytes at diagnosis (30)

GCA, giant cell arteritis; TAK, Takayasu arteritis; IL, interleukin; TNF, tumor necrosis factor; GM-CSF, granulocyte-macrophage colony-stimulating factor; JAK, Janus kinase; IFN, interferon.

**Table 2:** Molecular profiles in mouse models of large vessel vasculitis associated with treatments.

	Human artery engraftment mice	IL-1Ra deficient mice
IL-6 receptor antagonist		• Blockade of IL-6 signaling had limited therapeutic effect on inflammation (31, 32)
TNF-α receptor antagonist		• Reduced aortitis and arthritis in the absence of TNF-α (32, 33)
IL-1 receptor antagonist		• Increased transduction of IL-1 signaling activated CD4+ T cells and led to the migration of γδ T cells (34)
GM-CSF receptor antagonist		
JAK inhibitors	• Reduced infiltration of T cells and macrophages (35)	

GCA, giant cell arteritis; TAK, Takayasu arteritis; IL, interleukin; TNF, tumor necrosis factor; GM-CSF, granulocyte-macrophage colony-stimulating factor; JAK, Janus kinase.

### 2.1 IL-6 receptor antagonist

In LVV, IL-6 is produced by lymphocytes, macrophages, and neutrophils and plays a critical role in the pathogenesis of the disease (4, 39–41). Clinical trials in GCA and TAK have revealed that tocilizumab (TCZ), an IL-6 receptor antagonist, induces sustained remission, prolongs relapse time, and reduces the rate of new vascular events compared to a placebo. At the same time, the effect was more pronounced in patients with GCA than in those with TAK (42–47). TCZ inhibits antibody production by B cells in general (48), and normalized the imbalanced proportion of M0-like monocytes, activated/resting NK, Treg, Tfh, and Th17 cells in LVV (7, 21). In addition, TCZ reverses the glycolysis and calcium signaling abnormalities observed in Treg dysfunction in patients with GCA (8). However, it is important to note that TCZ has less effect on the proportion of Th1 and CD8 T cells in the circulation, and that Th1- and CD8-mediated inflammation may persist under TCZ treatment, particularly in TAK (10, 11). Th1- and CD8-mediated interferon (IFN)-γ responses are observed in TAK rather than in GCA (2–5). IFN-γ promotes proinflammatory response by exploring Th1 differentiation, cytotoxic T cell proliferation, and M1 macrophage polarization (49, 50). The incomplete response of TCZ in TAK pathogenesis is highlighted in IL-1Ra-deficient mice, where inflammation persists in IL-6 -/- mice and in those treated with TCZ, but is suppressed in *Tnfsf1a* (TNF-α) -/- mice (31–33).

### 2.2 TNF-α receptor antagonist

Available evidence suggests that both TNF-α and IL-6 play important roles in LVV pathogenesis, but their contributions may differ in GCA and TAK. TNF-α inhibition-based treatments such as infliximab, adalimumab, etanercept, and certolizumab have been shown to be effective in the treatment of TAK (51–53), suggesting that TNF-α is a substantial mediator in the pathogenesis of this disease. Conversely, clinical trials of TNF-α antagonists have failed to demonstrate efficacy in GCA treatment (22–24). Studies in IL-1Ra-deficient mice also support the association between TNF-α and TAK progression, as the mice exhibited significantly reduced aortitis and arthritis in the absence of TNF-α (32, 33). TNF-α acts in conjunction with IFN-γ to stimulate macrophages and induce the production of monocyte chemotactic proteins, particularly CCL2, which recruits monocytes expressing CCR2 to form multinucleated giant cells that are characteristic of LVV (26, 27, 54–56). Elevated CCL2 serum levels after treatment with GC suggest that CCL2 may contribute to treatment failure in LVV (57). At the transcriptome level, TNF-α inhibition, like TCZ, could improve the residual gene signature compared to GC monotherapy (25, 58). Methylome profiling of LVV patients has also revealed novel pathways in the disease pathogenesis (59); however, there is limited data associated with TNF-α inhibition. Further secondary analyses of immunocellular dynamics are required as evidence for the efficacy of TNF-α inhibition in TAK accumulates.

### 2.3 IL-1 receptor antagonist

IL-1 β, like IL-6 and TNF-α, is highly expressed in the inflamed arterial walls of patients with LVV, and may play a role in the disease pathogenesis (39, 40). Whole blood transcriptome gene expression analysis revealed that the molecular pathway related to IL-1 was significantly upregulated in patients with LVV compared to healthy controls. This correlated with the positron emission tomography (PET) vascular activity score, a disease extent score based on the distribution of affected arteries (25). Increased transduction of IL-1 signaling activates CD4+ T cells. Specifically, activated CD4+ T cells migrate CCR2+ γ delta T cells toward CCL2+ inflammatory tissues (34). Considering that IL-1Ra deficient mice develop TAK-like aortitis, inhibition of IL-1 signaling may be a promising approach for the treatment of LVV. While clinical trials evaluating IL-1 receptor antagonists in the LVV are ongoing, previous reports have shown that anakinra is effective in patients with refractory GCA, with improvement in inflammatory biomarker levels and symptoms, and resolution of arterial inflammation on PET-CT images (60, 61).

### 2.4 GM-CSF receptor antagonist

CD4+ T cells, macrophages, myofibroblasts, and endothelial cells produce GM-CSF. The GM-CSF receptor α is highly expressed in GCA-affected arteries, resulting in an autocrine amplification loop (28, 62). GM-CSF is a macrophage differentiation factor fundamentally involved in vascular inflammation. Recent data have shown that GM-CSF induces the differentiation of CD206+ MMP9+ macrophage, that play a role in arterial wall destruction during neo-angiogenesis or intimal hyperplasia (63–65). Using an ex vivo temporal artery culture model, GM-CSF increased macrophage activation, Th1 cell polarization, neo-angiogenesis, and tissue injury. Treatment with the GM-CSF receptor antagonist, mavrilimumab, reduced CD16- and CD3ϵ- positive cell infiltration and downregulated key molecules involved in T cell activation and differentiation (28). Among T cells, mavrilimumab decreased Th1 differentiation by reducing TNF-α and IFN-γ, while a direct effect on Th17 differentiation was not assessed (28, 55). In a recent phase 2 clinical trial, mavrilimumab demonstrated superiority over placebo in analyses of the time to flare and sustained remission in patients with GCA (66).

### 2.5 JAK inhibitors

As described previously, the pathogenesis of LVV involves multiple cytokines, including IL-6, TNF-α, IL-1, GM-CSF, and IFN-γ. Inhibition of these cytokines or agents that inhibit subsequent cellular signaling pathways may effectively treat LVV. Although JAK/STAT signaling and type I IFN signatures were not identified as distinct pathways using whole blood RNA sequencing from patients with GCA-dominant LVV, one of the IFN signature genes, *APOBEC3A*, was elevated at diagnosis and was associated with disease relapse (25). Pathways associated with JAK/STAT signaling and the type I IFN signature are enriched in CD4 and CD8 lymphocytes in the TAK and the aorta of LV-GCA (29, 30). Type I IFN transcripts were also shown to increase in the vessel walls of immunodeficient mice trans-engrafted with inflamed temporal arteries from patients with GCA (67). In an experimental animal model of LVV, inhibition of JAK1/3 effectively suppressed arterial wall lesioned T cells and inhibited macrophage infiltration and growth factor production, resulting in reduced neo-angiogenesis and intimal hyperplasia (35). JAK inhibitors, including baricitinib (JAK1/2 inhibitor) and tofacitinib (JAK1/2/3 inhibitor), have recently shown efficacy in pilot studies in patients with LVV (68, 69). Considering that blocking JAK1/2 may promote upregulation of Th17- and CD206+ macrophage-mediated inflammatory response (50, 70), investigation of the clinical efficacy depend on targeted JAK isoform selectivity is anticipated.

## 3 Discussion

We investigated alterations in the molecular profiles of patients with LVV and in mouse models of LVV associated with the following treatments: IL-6 receptor antagonist, TNF-α receptor antagonist, IL-1 receptor antagonist, GM-CSF receptor antagonist, and JAK inhibitors. Other potential therapies, including CTLA4-Ig (abatacept), anti-IL-17A antibody (secukinumab), anti-IL-12/23p40 antibody (ustekinumab), and anti-CD20 antibody (rituximab), were outside the scope of this study. Our study demonstrated that bDMARDs and JAK inhibitors improved the levels of dysregulated molecular profiles compared to GC monotherapy. These results are consistent with a study in patients with rheumatoid arthritis, which revealed that treatment with bDMARDs normalized the molecular signature to a greater extent than methotrexate monotherapy (71). Using bDMARDs and JAK inhibitors may induce deep molecular remission in various inflammatory connective tissue diseases (72–74).

CD4+ and CD8+ T cells play central roles in the pathogenesis of LVV (75, 76). Recent evidence suggests that Th1- and CD8-mediated inflammation may be less responsive to TCZ treatment, whereas the molecular signatures of Th17, Tfh, and Treg cells are improved. The incomplete response of IL-1Ra deficient mice to TCZ suggests a limitation of this treatment. Based on clinical evidence which demonstrated that the effect of TCZ is modest in TAK but dramatic in GCA, it is inferred that Th1-and CD8-mediated inflammation may be dominant in TAK. Therefore, targeting the Th1- and CD8-IFN-γ axis may be an important therapeutic strategy in TAK. Based on this evidence, TNF-α receptor antagonists, IL-1 receptor antagonists, GM-CSF receptor antagonists, and JAK inhibitors are potential therapies that may ameliorate residual inflammation in the LVV.

In this study, we reviewed the alterations in molecular profile of LVV. Evidence from human and murine studies has revealed changes in the immune profiles of the LVV following treatment with bDMARDs and JAK inhibitors. Despite treatment with bDMARDs and JAK inhibitors, residual immune cell activation was observed, which contributed to immune cell infiltration and damage to large arteries, resulting in arterial stenosis, aneurysm, and potentially life-threatening complications. Further studies are required to elucidate the molecular mechanisms underlying LVV.

## Author contributions

KM drafted the manuscript. All authors contributed to the article and approved the submitted version.
